# Biomimetic Nanotechnology for SARS-CoV-2 Treatment

**DOI:** 10.3390/v15030596

**Published:** 2023-02-21

**Authors:** Shuo Li, Xue Liu, Gang Liu, Chao Liu

**Affiliations:** 1 State Key Laboratory of Molecular Vaccinology and Molecular Diagnostics, National Institute of Diagnostics and Vaccine Development in Infectious Diseases, Center for Molecular Imaging and Translational Medicine, School of Public Health, Xiamen University, Xiamen 361102, China; 2 State Key Laboratory of Cellular Stress Biology, Innovation Center for Cell Biology, School of Life Sciences, Xiamen University, Xiamen 361102, China

**Keywords:** SARS-CoV-2, nanotechnology, nanodecoys, vaccine

## Abstract

More than 600 million people worldwide have been infected with severe acute respiratory syndrome coronavirus 2 (SARS-CoV-2), resulting in the pandemic of coronavirus disease 2019 (COVID-19). In particular, new waves of COVID-19 caused by emerging SARS-CoV-2 variants pose new health risks to the global population. Nanotechnology has developed excellent solutions to combat the virus pandemic, such as ACE2-based nanodecoys, nanobodies, nanovaccines, and drug nanocarriers. Lessons learned and strategies developed during this battle against SARS-CoV-2 variants may also serve as inspiration for developing nanotechnology-based strategies to combat other global infectious diseases and their variants in the future.

## 1. Introduction

The COVID-19 virus has already infected millions of people worldwide. The reason for this concerning situation is that many patients are contagious without showing any apparent symptoms. The government had to impose quarantine measures to prevent the virus’s spread. A triumph of speed and innovation, the first approved vaccines against SARS-CoV-2 have been developed by the German biotechnology company BioNTech, working with Pfizer, and by the American firm Moderna. Only sixty-three days after sequence selection, Moderna initiated a Phase I clinical trial for a lipid nanoparticle (LNP)-based mRNA vaccine (mRNA-1273). According to materials physicist Richard Jones, nanomedicine has entered its golden age.

SARS-CoV-2 is a 28-kilobase single-stranded positive RNA virus that undergoes two single-letter mutations per month [1], a relatively slow rate compared with other RNA viruses due to its proofreading capabilities. However, as a result of its rapid spread, more than 4000 variants have been reported so far [2]. A major concern regarding variants is their potential to impair immunity created by vaccines or previous infections. Although most variant strains contain several mutations, the most studied and concerning mutations are located in the SARS-CoV-2 spike (S) protein. S protein has more than 1200 amino acids, but only 25 are involved in the interaction between its receptor-binding domain (RBD) and the host cell’s angiotensin-converting enzyme 2 (ACE2) receptor. RBD mutations have the greatest impact on the ability of antibodies to neutralize virus infection [3]. Nanotechnology has contributed to the solution of this issue in many ways. For example, biomimetic nanotechnology is used to interfere with the interaction between the S protein and the ACE2 receptor of the host cell. Additionally, the use of nanoparticles (NPs) can enhance the stability of cargo storage such as DNA, RNA, proteins, or synthetic substances and protect them from degradation [4]. NPs can penetrate biological barriers to reach their target cells. Due to their similar size to viruses, NPs can simulate some of the behaviors of viruses in vivo. In addition to antigens, NPs can also deliver adjuvants to prime the immune system. In conjunction with vaccines, adjuvants assist in boosting immune responses by activating additional molecular receptors, which primarily recognize pathogens and danger signals. This co-delivery can help limit off-target effects by targeting antigens and adjuvants only to antigen-presenting cells (APCs) that have taken up the antigen [5]. Due to the targeted delivery of adjuvants, the amount of antigen required for immune protection can also be reduced, resulting in a dose-sparing effect [5]. This effect would be beneficial financially and practically in the current pandemic due to the enormous number of doses required. In this review, we aim to summarize the nano-based strategy to combat SARS-CoV-2, which will help public health departments and researchers make decisions and take measures to deal with SARS-CoV-2 and its fatal variants.

## 2. Nanotechnology Solutions to the Challenge of SARS-CoV-2

### 2.1. Nanotechnology for Treating SARS-CoV-2 Infection

**Figure 1:**
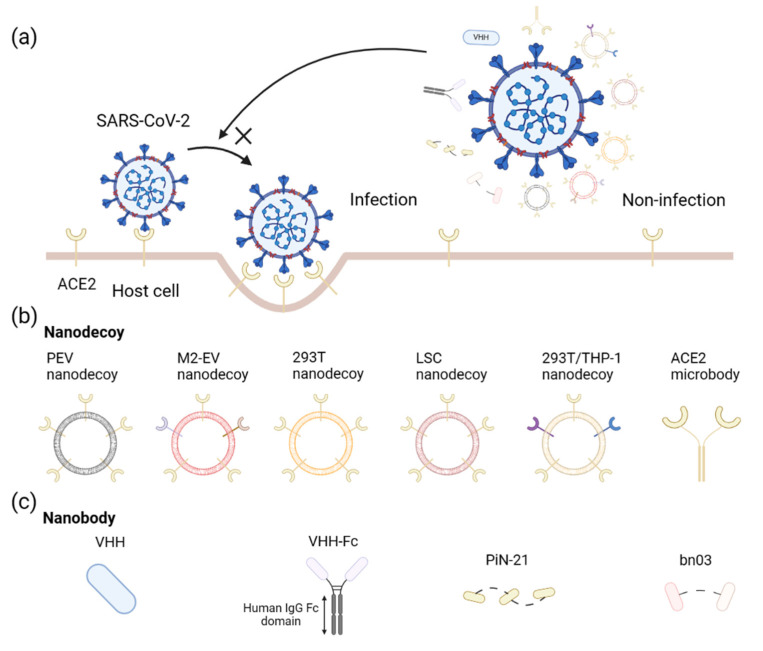
Nanodecoys and nanobodies targeting the S protein of SARS-CoV-2. (**a**) Nanodecoys and nanobodies prevent SARS-CoV-2 variants from binding to ACE2, protecting host cells from infection. (**b**) Nanodecoys: nanodecoys based on EVs and different membranes, including 293T [8], LSC [9], and 293T/THP-1 [10], an ACE-2 microbody containing two ACE2 receptors. (**c**) Nanobody: a single nanobody, nanobodies were conjugated to IgG Fc, a trimer containing three nanobodies, heterodimers of nanobodies.

**Figure 2:**
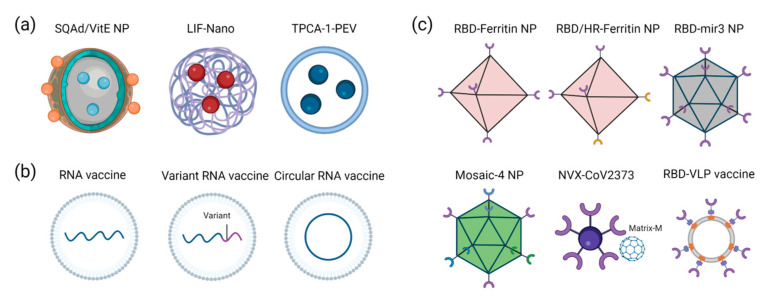
NP-based immunotherapy against SARS-CoV-2. (**a**) NPs for treating cell cytokine storm: an NP containing squalene, adenosine, and Vitamin E, encapsulated LIF within PLGA, platelet-derived extracellular vesicles loaded with TPCA-1. (**b**) Nucleic acid vaccines: NP-based RNA vaccine, variant RNA vaccine, and circular RNA vaccine. (**c**) Protein NP vaccines: the S protein fragments of the virus were attached to different NPs.

The SARS-CoV-2 virus infects host cells by binding to the ACE2 receptor expressed on their surface [6]. Mutations in the S protein of SARS-CoV-2 variants cause increased transmission and decreased antibody neutralization [7]. Therefore, targeting the S protein to inhibit the interaction with the ACE2 receptor may be the most direct and promising strategy (Figure 1). In most cases, nucleic acids, proteins, and drugs degrade rapidly once they enter the patient’s body. Nanotechnology offers a variety of solutions, including nanotechnology-based carriers for therapeutic drugs, nucleic acids, and protein NP vaccines (Figure 2).

#### 2.1.1. Nanodecoys

As a fundamental unit of biology, cells are capable of performing a wide range of functions, including interacting with the environment through membrane proteins. Biomimetic synthetic strategies are used to create biofunctionalized liposome-like nanovesicles (NVs) that can artificially display a variety of peptide ligands and targeting proteins [11]. This approach is inspired by the secretion process and natural cargo delivery functions of natural extracellular vesicles (EVs). Biomimetic NVs are self-assembled from broken cell membranes after ultrasonic treatment and endowed with specific biological functions of the source cells [12]. As a novel kind of drug carrier or technology, biomimetic NVs have been deeply studied in the treatment and diagnosis of diseases [11,13,14,15,16,17,18,19,20,21]. Cell membrane receptors are often crucial components of virus binding and entry and viable therapeutic targets for various infectious diseases. In our previous work, we developed a general method for producing nanodecoys that resist viruses through the overexpression of receptors through a natural biosynthesis process [21]. A widely used method for preparing nanodecoys targeting SARS-CoV-2 is constructing membrane NVs derived from ACE2-enriched cells. In a study, the nanodecoys, derived from the cellular membrane of genetically engineered 293T cells stably expressing hACE2, have been shown to have excellent neutralizing capabilities against both wild-type SARS-CoV-2 and the D614G variant pseudoviruses [8]. The inhalation of formulations containing nanodecoys and hyaluronic acid (HA) before infection successfully inhibited infection in an ACE2-expressing mouse model. The advantage of this inhalable formulation containing nanodecoys and mucoadhesive adjuvant HA is prolonged retention in the lungs, the primary organ of SARS-CoV-2 infection. However, despite HA being generally thought of as non-toxic and biocompatible, several studies have uncovered the immunogenicity of low-molecular-weight HA [22,23] and its role in macrophage activation [24]. Although in a preliminary stage, these results must be thoroughly evaluated to gain a better understanding of the potential of HA as an adjuvant. Similar nanodecoys have also been described to block infection by SARS-CoV-2 and its variants [25].

A study has shown that nanodecoys produced from human lung spheroid cells (LSCs) highly expressing ACE2 neutralize SARS-CoV-2 and protect the host from infection [9]. The LSC-nanodecoys were delivered to mice via inhalation therapy and remained in their lungs for more than 72 h. Furthermore, the study has shown that inhaling LSC-nanodecoys accelerated the clearance of SARS-CoV-2 mimics from the lungs, with no toxic side effects observed. Four doses of these nanodecoys delivered by inhalation to cynomolgus macaques challenged with live SARS-CoV-2 promoted viral clearance and reduced tissue damage associated with SARS infection. The post-infection therapeutic effect of LSC nanodecoys may be related to multiple mechanisms. Since not all viruses are internalized by host cells 1 or 2 days after virus exposure, nanodecoys can prevent the remaining viruses from entering cells. Furthermore, endocytosed nanodecoys can also bind intracellular viruses, reducing further infection. Using mixed cell membranes can further enhance the effectiveness of nanodecoys against SARS-CoV-2. In one study, 293T/THP-1 nanodecoys were developed by fusing membranes from 293T cells expressing genetically engineered ACE2 and human myelomonocytic THP-1 cells expressing cytokine receptors [10]. In this design, the nanodecoy’s ACE2 and cytokine receptors sequester SARS-CoV-2 and inflammatory cytokines, including interleukin-6 and granulocyte-macrophage colony-stimulating factor. It can reduce viral loads and effectively alleviate inflammatory reactions.

A recent study found that circulating ACE2-expressing EVs isolated directly from the plasma of patients with COVID-19 effectively inhibited SARS-CoV-2 infection in vitro [26]. EVs are vesicles with membrane structures released by cells. EVs were initially thought to be cellular debris and thus overlooked. However, many studies now demonstrate that EVs transfer diverse cargoes, including proteins, lipids, and nucleic acids [27], participate in intercellular communication in physiological activities and pathological changes, play an essential role in tumor metastasis and invasion, and can also serve as a necessary carrier of circulating biomarkers for disease diagnosis and prognosis. The research team found that ACE2-expressing extracellular vesicles (evACE2) exist in the blood of COVID-19 patients but not in healthy people, and the more severe the disease, the higher the level of evACE2 in the blood of COVID-19 patients, indicating that evACE2 is the body’s natural response to COVID-19 infection. Furthermore, evACE2 can effectively block the original coronavirus strain and mutant strains such as Alpha, Beta, and Delta. In addition, human peritoneal M2 macrophage-derived extracellular vesicles (M2-EVs) expressed ACE2 and thus could also serve as nanodecoys to prevent SARS-CoV-2 pseudovirus infection in vitro [28].

ACE2 receptor-modified decoy NPs have also been studied as a potential strategy against SARS-CoV-2. Recent studies have shown that soluble ACE2-related inhibitors, such as recombinant human ACE2, can partially block infection [29,30], but their short half-life [31] (<2 h in mice) limits their clinical application. However, a dimeric rhesus ACE2-Fc fusion protein had a plasma half-life of more than one week in mice [32]. An ACE2 microbody, consisting of two ACE2s fused to the fragment crystallizable (Fc) domain of IgG, inhibits the entry of the SARS-CoV-2 pseudotype virus in vitro [33]. The test of the half-life of the ACE2 microbody in vivo has not yet been performed, but the protein exhibited significant antiviral activity in tissue culture for several days, significantly longer than soluble ACE2. Moreover, intranasal co-administration of microbodies and the SARS-CoV-2 virus protected K18-hACE2 transgenic mice from SARS-CoV-2 infection-induced weight loss.

Several ACE2-based nanodecoys have entered clinical trials [34,35] and have been shown to be very effective against SARS-CoV-2 and its variants [36]. Further studies have shown that improving the half-life of nanodecoys or using the engineered ACE2 receptor with a higher affinity for the virus can further improve its performance [37,38,39,40]. Aside from acting as a receptor for SARS-CoV-2, ACE2 also regulates the renin-angiotensin system [41]. Thus, exogenous administration of ACE2-based nanodecoys may cause the down-regulation of the renin-angiotensin system and affect blood pressure and inflammation [42,43]. In addition, in most studies, the nanodecoys are administered during or before the infection with SARS-CoV-2. However, it is difficult to predict when an individual will be infected in a clinical setting. A study found that LSC nanodecoys [9] administered one or two days after virus exposure were still effective at sequestering the virus and attenuating lung injury, suggesting their potential as a post-infection treatment. Furthermore, the nanodecoy persists in the body for a short duration before being eliminated, thus requiring re-treatment upon re-exposure to the virus.

Monoclonal antibodies (mAbs) that target the S protein of SARS-CoV-2 have been widely utilized during the current COVID-19 pandemic [44]. However, due to the emergence of variants, most mAbs decreased in their neutralizing activity against Omicron, with only 3 out of 29 mAbs preserving their original potency [45]. The potential therapeutic effects of COVID-19 convalescent plasma are likely mediated by antibodies through direct viral neutralization and Fc-dependent functions such as phagocytosis, complement activation, and antibody-dependent cellular cytotoxicity [46]. In one study, COVID-19 convalescents had a humoral immune memory against the wild-type SARS-CoV-2 that could last up to two years [47]. However, plasma neutralization against various SARS-CoV-2 variants, including Delta, BA.1, BA.2, and BA.4/5, was significantly reduced, indicating a high risk of reinfection with emerging variants in these convalescents [47]. Despite their ongoing evolution, all SARS-CoV-2 variants gain entry into cells through the interactions of their S protein with the ACE2 receptor expressed on cells. Consequently, strong interactions between the ACE2 receptors of decoy NPs and the S proteins of the SARS-CoV-2 variants can guarantee that the sequestration of viruses is unaffected by virus mutations.

#### 2.1.2. Nanobodies

In contrast to conventional immunoglobulin G (IgG) antibodies that contain a heavy chain variable domain and a light chain variable domain, camelids can produce only one variable domain of a heavy chain of heavy-chain antibodies (VHH). In the absence of an effector domain, this single variable domain is referred to as a single-domain antibody, VHH, or nanobody. It has higher affinity and specificity than conventional antibodies [48]. Unlike IgG antibodies, nanobodies are small in size (about 15 kDa), highly soluble, and easy to bioengineer into bivalent or multivalent forms, making them suitable for low-cost and high-efficiency microbial production [49,50]. As biologics, nanobodies are particularly attractive for respiratory infections since they can be nebulized and administered directly to the site of infection via an inhaler [51]. In the early days of the outbreak, Cell published the first research results on nanobodies against SARS-CoV-2 [52]. Initially, the research aimed to develop nanobodies that neutralize SARS-CoV-1 and Middle East respiratory syndrome coronavirus (MERS-CoV). Wrapp alternately immunized a llama with the S proteins of SARS-CoV-1 and MERS-CoV and screened out nanobodies that could neutralize both viruses simultaneously. After the outbreak of the COVID-19 epidemic, a sequence comparison found that the gene sequence homology between SARS-CoV-1 S and SARS-CoV-2 S was very high, and the nanobody VHH-72 showed the ability to neutralize SARS-CoV-2 through preliminary screening, but its binding ability was weaker than that of SARS-CoV-1.

Using a humanized synthetic VHH library, researchers successfully screened neutralizing nanobodies against SARS-CoV-2 in May 2020 [53]. Through the S protein binding experiment with SARS-CoV-2, 69 specific nanobodies were screened out, 15 of which could block the binding of the S protein to the ACE2 receptor. Furthermore, the pairwise combination of VHHs showed a synergistic blocking effect, and when the nanobody was fused with human IgG-Fc, the blocking effect on S/ACE2 was further increased.

Using next-generation sequencing combined with mass spectrometry, Shi Yi’s group discovered over 8000 high-affinity anti-SARS-CoV-2 nanobodies in alpacas [54]; Nb21 has been designed in trimer form to enhance its antiviral activity. In collaboration with nebulization expert Dr. Doug Reed, the researchers developed an inhalable nebulizer for this nanobody, which they named Pittsburgh Inhalable Nanobody-21 (PiN-21) [55]. In hamsters, the researchers found that the amounts and activities of Pin-21 in bronchoalveolar lavage fluid were substantially lower 24 h after inhalation compared with 8 h after inhalation, possibly suggesting a more rapid clearance, while there was no difference in the levels of Pin-21 in the serum. This spray can treat severe cases of SARS-CoV-2 infections.

In recent months, the Omicron variant has spread throughout the world. This variant has more than 30 mutations in the S protein and 15 in the S-RBD protein. The mutations in the variant present a challenge to vaccine and antibody protection because it differs from other COVID-19 variants with no more than three mutations in the S-RBD protein. Recent research has demonstrated that two nanobodies (n3113v and n3130v) can neutralize the Omicron variant by binding to highly conserved regions on the RBD [56]. The two fully humanized nanobodies were linked together to obtain the nanobody bn03, which could efficiently neutralize various epidemic variants of SARS-CoV-2, including Omicron. In addition, a single-dose pharmacokinetics study in mice was conducted via inhalation using a high-pressure microsprayer. After inhalation, the mice were euthanized at designated time points to collect blood and lung samples, and the antibody concentration in plasma and lung was measured. The results showed that bn03 concentrations in the lung were significantly higher than those in the circulating blood after inhalation, and the half-life of bn03 in mice was found to be no more than 12 h.

Recently, researchers reported the isolation and characterization of a novel group of nanobodies from immunized alpacas [57]. These nanobodies, exemplified by the protein 3-2A2-4, have potency against Omicron subvariants BA.1, BA.2, BA.2.12.1, and BA.4/5, SARS-CoV-1, and key representative coronaviruses from bats and pangolins. To further study the in vivo protective effect of nanobody 3-2A2-4, researchers used a K18-ACE2 transgenic mouse model to conduct a preventive protection experiment. Nanobody 3-2A2-4 was administered intraperitoneally one day before challenge, and SARS-CoV-2 Omicron BA.1 and Delta live viruses were challenged nasally 24 h later, and the survival rate and body weight of mice were detected. The results showed that the 3-2A2-4 nanobody could effectively prevent Omicron and Delta live virus infection, prevent and reduce pulmonary tissue infection, protect pulmonary tissue from structural damage and an inflammatory response, and show excellent in vivo protective capacity.

Due to their small molecular weight and stable physical and chemical properties, nanobodies can be very effective and rapidly delivered to the respiratory tract, alveolar, and other foci of COVID-19 infection by inhalation. The nanobodies were found to be effective in inhibiting SARS-CoV-2 infection in both mild and severe mouse models, confirming their effectiveness and potential for clinical use. The study of nanobodies has some inherent limitations. Due to the differences in the immune response between alpacas and humans, the antibody responses and the epitopes recognized by the nanobodies may not be an accurate reflection of those in humans. Additionally, nanobody protection experiments were conducted in mice and hamsters, which may not be indicative of protection against SARS-CoV-2 infection in humans. It would be highly desirable to conduct future studies in humans to verify and validate the protection results.

#### 2.1.3. Nanocarrier for Treating Cytokine Storm

Some infectious diseases are associated with uncontrolled inflammation [58,59]. One of the main features of COVID-19 is that it triggers a cytokine storm in the body, which is caused by an exaggerated immune response and leads to acute deterioration of the patient’s health in a very short time [60]. This inflammatory storm is one of the leading causes of acute respiratory syndrome, often associated with multiple organ failure. Tocilizumab is a monoclonal antibody drug approved by the FDA for the treatment of COVID-19. It is used to reduce inflammation and improve symptoms in patients with severe cases of the virus [61]. It works by blocking interleukin-6, which is involved in the body’s inflammatory response to the virus. It is generally well-tolerated but can cause side effects such as nausea, headaches, and fatigue. Additionally, a clinical study revealed that tocilizumab was not effective in preventing intubation or death in moderately ill hospitalized patients with COVID-19 [62]. Therefore, further research on the use of nanotechnology to prevent excessive inflammatory responses is necessary.

Hyperimmune activity has been associated with several subpopulations of the immune system. A continuous positive feedback loop between pro-inflammatory signaling and oxidative stress often causes uncontrolled pro-inflammatory states. A number of challenges arise when it comes to inhibiting the pathological inflammatory response and the cross-talk between oxidative stress and inflammation [63]. However, although potent anti-inflammatory agents exist, such as corticosteroids, these have not been effective in acute inflammatory conditions such as sepsis due to the negative effects they have on tissue repair and the reported adrenocortical insufficiency common among sepsis patients [64]. Adenosine (Ad) is an endogenous anti-inflammatory agent that influences the function of inflammatory cells by interacting with specific receptors on the cells [65]. The endogenous purine Ad and Ad receptor agonists have shown promise in reducing inflammation [65,66]. However, their systemic administration results in rapid clearance [67] and unacceptable medical side effects due to untargeted activation of their cognate receptors [68,69]. As a result of the evolution of systemic inflammatory insults, the innate immune system’s initial response is transferred from plasma to tissue and cells. This results in disturbed signaling, dysfunction in cells, and organ failure. The best way to combat such processes is to target them at the sites of inflammation. As well, antioxidant supplementation failed to scavenge reactive species during acute inflammation due to poor pharmacodynamics and poor tissue penetration [70]. In recent years, multidrug treatments using hydrocortisone in combination with antioxidants have been shown to be a promising way to reduce uncontrolled inflammation by simultaneously inhibiting pro-inflammatory cascades and scavenging reactive oxygen species [71]. So far, however, most antioxidants used in this context act predominantly in plasma. This is useful during the initial hyperinflammatory stages of the body’s response, but it has limited effectiveness in inhibiting the pathological redox cycles happening inside cells and tissues [70]—as plasma antioxidant levels do not correlate well with intracellular antioxidant levels [72]. To address these issues, a targeted approach was taken to treat lethal hyper-inflammation associated with SARS-CoV-2 infection using an NP containing squalene, adenosine, and vitamin E (SQAD/VitE) [73]. SQAd/VitE NPs present a promising therapeutic intervention that overcomes the limitations of both conventional Ad therapy and antioxidant therapy because they can target and deliver Ad directly to tissue foci of acute inflammation and react with intracellular reactive species at the target site.

Leukemia inhibitory factor (LIF), secreted by mesenchymal stem cells (MSCs) [74], can be used against cytokine storms. However, its potency or production must be increased several-fold to cope with the response generated during COVID-19. A nano-technologically synthesized substitute of LIF, LIF-Nano, which encapsulated LIF within polymer poly (lac-tide-co-glycolide) (PLGA), is almost 1000 times more potent than its counterpart, which can protect the lungs from hyper-inflammation [75]. Platelet-derived extracellular vesicles (PEVs) have been engineered to deliver pneumonia-targeted drugs based on their intrinsic affinity for inflammation [76]. In a mouse model of acute lung injury, PEVs generated from activated platelets can selectively target pneumonia. As a result of loading the PEVs with [5-(p-fluorophenyl)-2-ureido] thiophene-3-carboxamide (TPCA-1), which can inhibit the production of inflammatory factors, the therapeutic benefits were significantly improved by inhibiting the infiltration of pulmonary inflammatory cells as well as calming the local cytokine storm compared with free drug therapy. Macrophages are one of the innate immune cells involved in host defense, widely present in all tissues, and their phenotype switching plays an essential role in maintaining immune homeostasis. M2 macrophages mainly play roles in immune regulation and tissue repair. Recent studies have found that M2-EVs can be used as a multi-targeted nanomedicine for treating cytokine storms caused by pathogen infections [28].

### 2.2. Nanotechnology for Preventing SARS-CoV-2 Infection

#### 2.2.1. mRNA LNP Vaccines

Despite the potential of mRNA for gene therapy and vaccine development, clinical translation is limited by drug delivery. Since in vivo injection of naked mRNA results in rapid endonuclease-mediated degradation, novel approaches are required to improve the delivery of nucleic acid vaccines. LNPs serve as a viable delivery system for enhancing the efficacy of RNA vaccines and can introduce genes into cells and protect them from degradation in vivo.

In recent years, a large body of preclinical data has been generated on mRNA vaccines, and several clinical trials have been conducted in humans. Medical history records the fastest development of mRNA vaccines by Pfizer-BioNTech (BNT162b2), Moderna (mRNA-1273), and CureVac [77,78,79]. Injection of mRNA-LNPs leads to internalization by antigen-presenting cells, where ribosomes translate them into S-protein antigens [80]. Subsequently, the S-protein antigen is degraded into antigenic fragments to activate CD8+ T cells; meanwhile, endocytosis of the secreted S protein antigen activates CD4 T cells and B cells to produce neutralizing antibodies. Much of the discussion concerning vaccine efficacy revolves around T follicular helper cells (TFH). It is a distinct subset of CD4+ T cells that are required for the development of germinal center (GC) responses, immunoglobulin class switching, affinity maturation, and persistence of long-term B-cell memory [81]. Therefore, antigen-specific TFH cells are critical in generating a sustained, broad-based protective antibody response. For mRNA vaccines, the production of the antigenic protein is transient, and the mRNA is then naturally degraded in the cell. Modified nucleosides can reduce the immunogenicity of mRNA and improve translation efficiency [82]. In one study, nucleoside-modified mRNA-LNP vaccines induce high levels of antigen-specific TFH and GC B cells [83].The results revealed that mRNA-LNP vaccinations outperformed inactivated virus vaccines, adjuvant protein vaccines, and live pathogen infections regarding TFH cell abundance and induction of long-lived plasma and memory B cells [84]. It has been shown that this results from both potent and persistent antigen production and adjuvant effects induced by encoded mRNA and LNPs [85]. Ionizable lipids in LNP formulations drive early cytokine production in draining lymph nodes after intramuscular injection, most notably through interleukin-6 signaling. This rapid rise in interleukin-6 concentrations is critical for the induction of downstream effectors that drive TFH and GC responses [85]. Although the mRNA in the LNP has been modified to reduce pathogen-recognition receptor sensing, it still generates a cytokine response that partially supports the generation of TFH cells. The initial presentation of SARS-CoV-2 antigens by dendritic cells can be derived from either processed live virus antigens or antigens encoded in the vaccine mRNA. After infection with SARS-CoV-2, increased numbers of activated TFH cells were detected in the blood of non-severely infected patients and the lymph nodes of rhesus monkeys. A significant correlation was found between high levels of activated circulating TFH cells and low disease severity. The antigen-specific circulating TFH cells persisted for at least six months after infection [81].

Despite their high effectiveness against the original SARS-CoV-2 strain, these mRNA vaccines were insufficient against emerging SARS-CoV-2 variants due to mutations in the viral S protein. Therefore, several strategies have been developed to update current mRNA-LNP vaccines or generate new mRNA-LNP vaccines to increase neutralizing antibodies that bind to the mutated S protein and neutralize SARS-CoV-2 variants. Since decreased vaccine-induced immunity is one of the primary reasons for reduced vaccine efficacy against SARS-CoV-2 variants, boosting vaccine-induced immunity through booster doses of vaccines is the most straightforward and effective way to address this problem. Consequently, the Food and Drug Administration approved a booster dose of the mRNA vaccine from Pfizer or Modena for adults. Recent studies have shown that administering a third booster dose of Pfizer’s BNT162b2 or Moderna’s mRNA-1273 vaccine to solid organ transplant recipients significantly improved the immunogenicity of SARS-CoV-2 vaccines [86,87]. Another study found that a third dose of the BNT162b2 vaccine significantly increased the neutralization of SARS-CoV-2 and its variants [88]. Even with the current prevalence of highly infectious variants of Omicron, booster doses of mRNA-1273 or BNT162b2 vaccines may still induce potent neutralizing antibodies to Omicron and protect against it, despite reduced efficacy [89,90,91]. Another strategy proposed by Moderna to address SARS-CoV-2 variants is the development of novel mRNA vaccines [92]. Thus, a modified version of the prototype mRNA-1273 vaccine, named mRNA-1273.351, which contains the gene sequence for the S protein of the Beta variant, has been developed [93].

The storage temperature for mRNA vaccines must be extremely low, which limits their widespread use [94]. To address this issue, LNP-based circular RNA vaccines have been developed [95]. Since circular RNA vaccines have a structure that nucleases cannot easily degrade, they exhibit higher stability than linear mRNA vaccines. A circular RNA vaccine containing the SARS-CoV-2 variants RBD (Delta and Omicron) was administered to mice and monkeys and showed effective protection against the variants [95]. Freeze-drying of mRNA-LNPs is another possible solution, although some argue that this process is too expensive and time-consuming [96].

#### 2.2.2. Protein NP Vaccines

In addition to nanodecoys and nucleic acid vaccines, protein-NP vaccines are also being investigated against SARS-CoV-2 variants. There are four major structural proteins encoded by SARS-CoV-2: S, membranes, envelopes, and nucleocapsids [97]. The S protein consists of S1 and S2 subunits, and the heptapeptide repeats (HR) of the S2 subunit play an essential role in the fusion of the viral membrane and the host cell membrane. Using the SpyTag-SpyCatcher system, the researchers developed three different RBD-conjugated NP vaccines: RBD-Ferritin (24-mer), RBD-mi3 (60-mer), and RBD-I53-50 (120-mer) [98]. The three NP vaccines maintained the spatial conformation of RBD and significantly enhanced protein stability and antigenicity. Vaccines with these properties exhibit strong immune effects that are 120 times greater than those with monomeric RBD antigens. In addition, another protein NP vaccine simultaneously links the RBD and the HR of the S protein to the surface of ferritin [99]. Compared with RBD or HR monomer immunization, the antigen display technology of NPs more efficiently presents the immunogen to the body’s immune system. This can maintain the original antigenic conformation of RBD and HR to a great extent. The newly developed NP vaccine induced a high titer of neutralizing antibodies against SARS-CoV-2 and could potentially neutralize a variety of other coronaviruses, showing a certain cross-protective effect.

There will always be a step or two behind the evolving virus when the COVID-19 vaccines are updated. Scientists hope to develop a vaccine that can be broadly protective against future SARS-CoV-2 variants and even other coronaviruses associated with it. A quadrivalent mosaic NP vaccine displaying S proteins from a prototype SARS-CoV-2 and three different variants was designed to address this issue [100]. The mosaic NPs elicit similar or superior neutralizing antibodies against variant strains when tested on mice and non-human primates, with only a small reduction in neutralization titers against the ancestral strain. A notable feature of this vaccine is its ability to prevent infection with the prototype and B.1.351 strains in mice. The results demonstrate the feasibility of developing multivalent vaccines against pandemic and pre-emergent SARS-CoV-2 strains. Recently, Cohen et al. developed mosaic NPs displaying the RBDs from SARS-CoV-2 and seven other animal sarbecoviruses [101]. Despite the absence of the SARS-CoV-1 RBD on mosaic-8 RBD NPs, mosaic nanoparticles protected against both SARS-CoV-2 and SARS-CoV-1 challenges in animal models. By contrast, homotypic SARS-CoV-2 RBD NPs were only effective against SARS-CoV-2 infections.

On 13 July 2022, Novavax’s novel coronavirus recombinant protein vaccine, NVX-CoV2373, received an Emergency Use Authorization from the FDA. The process of creating the vaccine begins with the identification of the antigen S that can stimulate an immune response against the virus [102]. This antigen is then modified and inserted into a baculovirus, which is used to infect Sf9 cells. The baculovirus replicates inside the cells, and the recombinant antigen gene enters the cell nucleus and is transcribed into mRNA. The Sf9 cells then use their natural machinery to translate the mRNA and produce large quantities of the recombinant antigen protein. These proteins are harvested from the surface of the cells, purified, and arranged around a nanoparticle core. In order to create an effective vaccine, the recombinant antigen protein nanoparticles are mixed with the Matrix-M adjuvant. Nanoparticles with S proteins are a key component of the Novavax vaccine. The S proteins act as a signal, while the Matrix-M adjuvant boosts the signal. Together, these two components are essential for the vaccine’s effectiveness. A two-dose regimen of the NVX-CoV2373 vaccine was found to be highly effective in protecting adult participants from SARS-CoV-2 infection, with an efficacy rate of 89.7%. Additionally, it showed high efficacy against the B.1.1.7 variant [103].

A virus-like particle (VLP) is a nanostructure that is self-assembled and incorporates essential viral structural proteins [104]. VLP has the same molecular and morphological characteristics as real viruses, but it is not infectious or replicable since it does not contain genetic material. A SARS-CoV-2 VLP vaccine that contains the four structural proteins of SARS-CoV-2 has been produced in suspension-adapted HEK293 cells [105]. Alum-adsorbed, K3-CpG ODN-adjuvanted VLPs could induce high titer of anti-S, anti-RBD, anti-N IgG, and neutralizing antibodies in mice, rats, and ferrets [105]. This VLP vaccine could promote multifunctional Th1-biased T-cell responses and show immunoprotective effects against a live SARS-CoV-2 challenge in vaccinated mice. Recently, a cost-effective RBD-VLP vaccine for SARS-CoV-2 produced in yeast has been developed [106]. The modular design of protein antigens combined with existing large-scale manufacturing capacity in yeast makes up a vaccine platform that could keep up with an evolving COVID-19 pandemic.

## 3. Conclusions

SARS-CoV-2 caused the current pandemic, and emerging SARS-CoV-2 variants resulting from persistent mutations in the S protein of SARS-CoV-2 exacerbated and prolonged the pandemic. Targeting mutant S proteins of SARS-CoV-2 variants through nanotechnology-based strategies holds great promise for combating SARS-CoV-2 variants. This latest variant of Omicron still infects host cells via the ACE2 receptor despite having more mutations than other variants. The development of ACE2-based nanodecoys is considered a promising strategy for targeting variant S proteins since all variants have a high binding affinity to the ACE2 receptor, regardless of their persistent mutations. Nanobodies offer another way to target mutant S proteins, especially when vaccine-elicited neutralizing antibodies are insufficient due to impaired immune responses. Although many monomeric antibodies that neutralize SARS-CoV-2 cannot neutralize emerging SARS-CoV-2 variants, multivalent displays of these antibodies on nanoplatforms can significantly promote neutralizing activity. The use of vaccines to induce a specific immune response is another effective way to target mutant S proteins and inhibit virus infection. A wide variety of vaccines have been developed using various technologies, some of which have shown high protective efficacy and have been deployed worldwide. However, the protective efficacy of these vaccines has declined when faced with emerging SARS-CoV-2 variants. The development and application of nanotechnology have provided many solutions for enhancing vaccine efficiency, promoting therapeutic drug delivery, and protecting individuals from SARS-CoV-2 variants. It is believed that the progress and innovation of nanotechnology will provide many ways to accelerate the end of the epidemic.

## Data Availability

All data is referred to in the references.

## Abbreviations

**Abbreviation**	**Definition**
SARS-CoV-2	Severe acute respiratory syndrome coronavirus 2
COVID-19	Coronavirus disease 2019
LNP	Lipid nanoparticle
S	Spike
RBD	Receptor-binding domain
ACE2	Angiotensin-converting enzyme 2
NPs	Nanoparticles
APCs	Antigen presenting cells
NVs	Nanovesicles
EVs	Extracellular vesicles
HA	Hyaluronic acid
LSCs	Lung spheroid cells
evACE2	ACE2-expressing extracellular vesicles
M2-EVs	M2 macrophage-derived extracellular vesicles
Fc	Fragment crystallizable
mAbs	Monoclonal antibodies
IgG	Immunoglobulin G
VHH	Variable domain of heavy chain of heavy-chain antibody
MERS-CoV	Middle east respiratory syndrome coronavirus
PiN-21	Pittsburgh inhalable nanobody-21
Ad	Adenosine
SQAD/VitE	An NP containing squalene, adenosine, and vitamin E
LIF	Leukemia inhibitory factor
MSCs	Mesenchymal stem cells
PLGA	Polymer poly(lac-tide-co-glycolide)
PEVs	Platelet-derived extracellular vesicles
TPCA-1	[5-(p-fluorophenyl)-2-ureido] thiophene-3-carboxamide
TFH	T follicular helper cells
GC	Germinal center
HR	Heptapeptide repeats
VLP	Virus-like particle
